# Betel nut chewing and incidence of newly diagnosed type 2 diabetes mellitus in Taiwan.

**DOI:** 10.1186/1756-0500-3-228

**Published:** 2010-08-17

**Authors:** Chin-Hsiao Tseng

**Affiliations:** 1 National Taiwan University College of Medicine, Taipei, Taiwan; 2 Division of Endocrinology and Metabolism, Department of Internal Medicine, National Taiwan University Hospital, Taipei, Taiwan; 3 Division of Environmental Health and Occupational Medicine of the National Health Research Institutes, Taipei, Taiwan

## Abstract

**Background:**

Betel nut chewing is associated with type 2 diabetes mellitus (T2DM) in a recent prevalence study in Taiwan. The present study further investigated its link with the incidence of newly diagnosed T2DM during the years 1992-1996.

**Methods:**

Population-based datasets of a sample of 93,484 out of 256,036 diabetic patients from 66 medical settings using the National Health Insurance scheme covering > 96% of the population, published population prevalence of betel nut chewing and the governmental census of national population were used for calculation of odds ratios, incidence rates and incidence rate ratios between chewers and never-chewers in the male population for the year 1992 to 1996.

**Results:**

Ever chewers among the diabetic patients were younger, more obese and had higher prevalence of parental diabetes than never-chewers (all *p *values < 0.001). Odds ratios for T2DM for ever chewers vs. never-chewers in the age of < 40, 40-49, 50-59, 60-69 and ≥70 years were 1.06 (0.92-1.23), 1.60 (1.45-1.76), 2.12 (1.88-2.39), 3.58 (3.10-4.13) and 7.14 (5.47-9.31), respectively. In 1996, incidence rates (per 100,000 population) in the respective age groups were 19.1, 251.5, 567.3, 721.7 and 971.4 for never-chewers; and were 30.2, 520.9, 2566.9, 11672.8 and 630.3 for ever chewers. The respective incidence rate ratios were 1.58, 2.07, 4.52, 16.17 and 0.65. The age-specific incidence rates and rate ratios were relatively consistent from 1992 to 1996. The differences in obesity and parental diabetes between ever chewers and never-chewers were mostly not statistically significant after age stratification, suggesting the link could not be attributed to these two factors.

**Conclusions:**

Chewing betel nut is associated with newly diagnosed T2DM, supporting the suggestion that the habit is diabetogenic.

## Background

Areca nut is the seed of the palm tree *Areca catechu*, which is the fourth most commonly used psychoactive substance, after caffeine, nicotine and alcohol [1]. Chewing of areca nut has always been referred to as 'betel nut chewing' in the English literature because areca nut is always consumed with the leaf of *Piper betle *[2]. Betel nut chewing is a common habit and a means of social interaction in Asia, particularly the South Pacific islands, Southeast Asia, Papua New Guinea, Bangladesh, Pakistan and India [1-4]. An estimated 600 million people are chewing betel nut worldwide [3].

Chewing of betel nut was forbidden in Taiwan during the Japanese reign more than 60 years ago [4]. But this habit has become popular during the past two to three decades [5]. The chewing population in Taiwan keeps on increasing, especially in the male sex of the younger generation [6,7]. In a population-based survey performed during the years 1999-2001 in Taiwan, the prevalence of betel nut chewing in the male population was as high as 14.3% [8]. In Taiwan, unripe areca nut is commonly chewed with a mixture of lime and the leaf or flower of the *Piper betle*, but without tobacco [4].

Betel nut chewing has been linked to a variety of health problems including oral lesions of leukoplakia, submucosal fibrosis, squamous cell carcinoma and periodontal disease [9], albuminuria in diabetic patients [10], disruption of gastric mucosal barriers [11], aggravation of asthma [12], induction of extrapyramidal syndrome [13], milk-alkali syndrome (in a case report) [14], induction of uterine cervical dysplasia [15], cancers of the esophagus [16] and liver [17], low birth weight of babies born to mothers chewing betel nut [18], predisposition to colonization of Helicobacter pylori in the digestive tract [19], hypertension [20], obesity [21], diabetes mellitus [8,22], and metabolic syndrome [23,24].

In the population-based study in Taiwan published recently, betel nut chewing is associated with a higher risk of type 2 diabetes mellitus (T2DM) [8]. However, this study analyzed prevalence data with estimated odds ratios of low strength of association ranging from 1.29 to 1.41 [8]. The odds ratios could underestimate the risk if the subjects chewing betel nut had a poorer prognosis due to dying from diabetes or other related cancers. Furthermore, the temporal correctness could not be discerned from the cross-sectional analyses of prevalence data. Therefore, further confirmation by incidence data is deemed more appropriate to investigate the strength of association and to discern a cause-effect relation. Taking into account the lower incidence of T2DM in the younger generations and the possible requirement of long-term exposure to betel nut chewing to develop T2DM, it is not easy to perform a longitudinal community follow-up study to compare the incidence of T2DM between people chewing and not chewing betel nut. However, incidence rates and their rate ratios between chewers and never-chewers in the whole population can be calculated if we have reliable data to estimate the numbers in the populations at risk and the incident cases of diabetes among the populations. The data extracted from an established nation-wide cohort of diabetic patients using the National Health Insurance (NHI) [25,26] together with the population-based estimates of the age-specific prevalence rates of betel nut chewing [8] and the complete population census data reported yearly by the government [27] provided a chance to calculate and compare the yearly incidence rates of T2DM in the populations chewing betel nut and those not chewing in specific subgroups by age. Therefore, the purpose of this study was to estimate the odds ratios and the yearly incidence rates of newly diagnosed T2DM from the year 1992 to 1996 (before the revision of the diagnostic criteria for diabetes proposed by the American Diabetes Association in 1997 [28]); and the incidence rate ratios between chewers and never-chewers to see whether a higher risk of T2DM in chewers could be consistently demonstrated, by using the above-mentioned population-based data in Taiwan. Because the male sex highly outnumbers the female in betel nut chewing in Taiwan [29] and the population-based age-specific prevalence rates of betel nut chewing are only obtainable for the male sex [8], this study assessed the association only in the male population.

## Methods

### Study Subjects

The study was approved by an ethics committee of the Department of Health of Taiwan. Because more than 96% (with the exemption of persons involved in military services and those subject to criminal sanction, etc.) of the total population of Taiwan has been covered by the compulsory NHI scheme since March 1995, almost all diabetic patients are seen in the NHI [25,26]. Therefore, the clinical settings' databases claiming for the NHI are appropriate to derive a national sample of diabetic patients. The assembly of such a national sample was described in detail elsewhere [25,26]. In brief, we identified a total of 256,036 diabetic patients from 66 hospitals and clinics located evenly throughout Taiwan. To create a cohort of 90,000 patients (approximately one-sixth of the estimated number of 540,000 diagnosed diabetic patients in Taiwan during the period) for long-term follow-up, one out of every two identified patients (i.e. 128,572 cases from the 256,036 patients) were randomly selected, assuming a response rate of 70%. The total number of diagnosed diabetic patients covered by NHI between July 1997 and June 1998 was 536,159 [30].

### Telephone interview

From March 1, 1995 to April 30, 2002, well-trained interviewers used a structured questionnaire for telephone survey. Researchers tried up to three times to reach subjects before giving up. The interviewers handed in the interviewed questionnaires every week and all returned questionnaires were checked by an assistant and then double-checked by the investigator.

The information extracted from the questionnaire for this study included the age at diabetes diagnosis, symptoms at diabetes onset, and treatment modality for the distinction between type 1 diabetes mellitus (T1DM) and T2DM, body height, body weight, betel nut chewing history (yes/no/quitted) and parental history of diabetes.

The classification of T1DM was based on either one of the following two criteria: 1) diabetic ketoacidosis at the diagnosis of diabetes mellitus; and 2) the patients required insulin injection within one year of diagnosis of diabetes mellitus. If a patient was not diagnosed as T1DM, he/she was viewed as a patient with T2DM. Patients identified as T1DM were not included into the present study.

### Statistical analyses

Betel nut chewers were classified as current chewers, ex-chewers and never-chewers. Because there were too few ex-chewers in stratification analyses and ex-chewers might have stopped chewing betel nut after the development of diabetes or related illness, ex-chewers and current chewers were pooled together as ever chewers in the calculations of prevalence and incidence rates of diabetes. Body mass index (BMI) was calculated from the body weight in kg divided by the squared body height in meters and obesity I and II were defined as a BMI ≥25 kg/m^2 ^and ≥30 kg/m^2^, respectively, as recommended for Asian populations [31]. The age at new diagnosis of T2DM was divided into < 40, 40-49, 50-59, 60-69 and ≥70 years for appropriate comparison with the age-specific prevalence of betel nut chewing reported by Tung et al. [8]. *P *< 0.05 was considered as statistically significant.

The baseline characteristics of age, BMI, obesity I, obesity II and parental diabetes between ever chewers and never-chewers were compared by Student's *t *test for continuous variables and by Chi-square test for categorical variables in all diabetic men and in the incident cases identified within the years 1992-1996, respectively.

The distribution of age-specific case numbers of all diabetic men by betel nut chewing was calculated and the prevalence rates of ever chewers between the diabetic patients from the present study and the referent study [8] were compared by Chi-square test. Odds ratios and their 95% confidence intervals for diabetic patients vs. the general population were estimated for ever chewers vs. never-chewers and for current chewers vs. never-chewers, respectively.

In Taiwan, a household registration system has been strictly enforced. Therefore, age- and sex-specific population numbers are very complete and accurate. The data for recent years are available as electronic files [27]. The age-specific mid-year population data of the male sex released yearly by the government [27] along with the age-specific prevalence rates of betel nut chewing among men in the population-based survey by Tung et al. [8] were used to calculate the numbers in the population who were betel nut ever chewers and never-chewers in specific age groups during the years 1992-1996. These yearly age-specific populations for ever chewers and never-chewers were used as denominators in the calculation of incidence rates of newly diagnosed T2DM for ever chewing and never-chewing populations, respectively. The data obtained from the interviewed diabetic cohort was used to calculate the age-specific incident case numbers of newly diagnosed T2DM with regards to betel nut chewing during the years 1992-1996. The incident numbers were multiplied by a correction factor of 5.74 when used as numerators for the calculation of incidence rates [26]. This figure was derived by dividing the total case number of diabetes using the NHI (536,159) [30] by the total case number receiving the interview in this study (93,484). The incidence rate ratios were derived by dividing the incidence rates in ever chewers by the corresponding rates in never-chewers. Because the incident rates were calculated based on large population data, confidence intervals were not estimated.

To examine whether obesity or parental diabetes could confound the effect of betel nut chewing, the prevalences of these variables were compared between ever chewers and never-chewers in the respective subgroups of age in different calendar years and Chi-square test was used for statistical test for the differences.

## Results

A total of 93,484 (response rate: 72.7%) cases completed the interview. Because the questions on betel nut chewing were added some time after the start of the interview, those interviewed earlier would not have data for betel nut chewing. After excluding patients with T1DM (*n *= 3,528), those with an unknown history of betel nut chewing (*n *= 8,730), and a further exclusion of female patients with T2DM (*n *= 44,000) there were a total of 37,226 male patients with T2DM included in this study. Among them 29,641 were never-chewers of betel nut and 7,585 were classified as ever chewers (4,294 current chewers and 3,291 ex-chewers).

Table 1 compares the baseline characteristics between the ever chewers and never-chewers in the incident cases during the years 1992-1996 and in all prevalent cases of T2DM. The chewers were significantly younger, more obese and had higher prevalence of parental diabetes.

**Table 1:** Baseline characteristics of type 2 diabetic men between ever chewers and never-chewers of betel nut for all patients and for incident cases during the year 1992 to 1996

Variables	Incident cases		All cases	
		
	Ever chewers	Never-chewers	Ever chewers	Never-chewers
*n*	3,453	12,565	7,585	29,641
Age, years	56.9 (10.9)	61.2 (11.8)*	57.3 (11.0)	62.4 (11.9)*
Body mass index, kg/m^2^	24.9 (3.6)	24.5 (3.4)*	24.7 (3.5)	24.4 (3.4)*
Obesity I	44.7	40.3*	42.9	38.4*
Obesity II	8.1	5.6*	7.1	5.3*
Parental diabetes	31.3	26.5*	30.4	26.8*

Data are expressed as mean (SD) or percentage.

Obesity I and II represent body mass index ≥25 and ≥30 kg/m^2^, respectively.

*P < 0.001 comparing ever chewers and never-chewers.

Table 2 shows the age distribution among all the men with T2DM identified in this study by betel nut chewing, the age-specific prevalence rates of ever chewers between the diabetic men and the referent general population [8], and the odds ratios and their 95% confidence intervals for T2DM for ever chewers vs. never-chewers and for current chewers vs. never-chewers. The age-specific prevalence rates of ever chewers and odds ratios of T2DM for ever chewers vs. never-chewers were all statistically significant except for the age group < 40 years. For current chewers vs. never-chewers, odds ratios were statistically significant for all age groups.

**Table 2:** Age-specific case numbers of all diabetic men by betel nut chewing in the present study, prevalence of betel nut ever chewers, and the estimated odds ratios for type 2 diabetes mellitus for ever chewers vs. never-chewers

Age (years)	Total	Current chewers	Ex-chewers	Never-chewers	Prevalence rate of ever chewers (%)		Odds ratio (95% confidence interval)	
						
					Present study	Referent study	Ever chewers vs. Never-chewers	Current chewers vs. never-chewers
< 40	1474	327	88	1059	28.2	26.9	1.06 (0.92-1.23)	1.53 (1.30-1.81)
40-49	5231	1064	497	3670	29.8	21.0*	1.60 (1.45-1.76)	2.03 (1.80-2.29)
50-59	8447	1366	869	6212	26.5	14.5*	2.12 (1.88-2.39)	2.31 (1.99-2.69)
60-69	11891	1142	1218	9531	19.8	6.5*	3.58 (3.10-4.13)	3.24 (2.66-3.93)
≥70	10183	395	619	9169	10.0	1.5*	7.14 (5.47-9.31)	6.29 (4.20-9.43)
Total	37226	4294	3291	29641	20.4	14.3*	1.53 (1.45-1.61)	1.60 (1.50 -1.71)

*P < 0.001 by Chi-square test comparing the prevalence rates of betel nut chewing between the diabetic patients in the present study and the general population in the referent study (reference [8])

Table 3 shows the age-specific incidence rates of newly diagnosed T2DM (per 100,000 population) and the incidence rate ratios for newly diagnosed T2DM for ever chewers vs. never-chewers in the years 1992 to 1996. It was evident that, except in the eldest population above 70 years of age, the incidence rates in ever chewers were higher than those in never-chewers in different age groups and incidence rate ratios increased with age, reaching a peak at the age of 60-69 years. These findings were consistent over the 5 years of observation.

**Table 3:** Age-specific incidence rates (per 100,000 population) and incidence rate ratios (RR) for newly diagnosed type 2 diabetes mellitus for ever chewers vs. never-chewers during the years 1992-1996

Year	Age (years)	Observed incident case		Incidence rate*		Incidence RR (Ever chewers vs. never-chewers)
				
		Ever chewers	Never-chewers	Ever chewers	Never-chewers	
1992	< 40	11	33	3.6	2.9	1.25
	40-49	66	152	229.1	89.6	2.56
	50-59	107	326	1145.6	241.3	4.75
	60-69	140	547	6997.8	423.5	16.52
	≥70	64	428	627.3	700.5	0.90
						
1993	< 40	19	39	6.2	3.4	1.83
	40-49	83	235	272.0	130.7	2.08
	50-59	209	494	2228.8	364.2	6.12
	60-69	216	774	10717.2	594.9	18.02
	≥70	96	661	882.8	1015.0	0.87
						
1994	< 40	31	68	10.1	5.9	1.71
	40-49	170	384	524.6	201.2	2.61
	50-59	259	652	2747.8	478.3	5.75
	60-69	259	951	12791.4	727.5	17.58
	≥70	99	790	853.3	1137.0	0.75
						
1995	< 40	48	126	15.7	11.0	1.43
	40-49	207	459	599.3	225.6	2.66
	50-59	241	682	2580.6	504.9	5.11
	60-69	206	877	10288.9	678.5	15.16
	≥70	84	668	680.9	904.2	0.75
						
1996	< 40	92	218	30.2	19.1	1.58
	40-49	193	549	520.9	251.5	2.07
	50-59	239	764	2566.9	567.3	4.52
	60-69	231	922	11672.8	721.7	16.17
	≥70	83	766	630.3	971.4	0.65

*The nominators for the incidence rates are the observed incident case number multiplied by a correction factor of 5.74 (please refer to the section 'Methods') and the denominators are the respective age-specific mid-year population of the male-sex in the specific year.

Table 4 compares the age-specific prevalence rates of obesity and parental diabetes between ever chewers and never-chewers in incident cases of newly diagnosed T2DM in 1992-1996. Except for 5 categories (indicated by asterisk), these prevalence rates did not differ significantly between ever chewers and never-chewers.

**Table 4:** Comparisons of age-specific prevalence rates of obesity and parental diabetes between ever chewers and never-chewers of betel nut in incident cases of newly diagnosed type 2 diabetes mellitus during the years 1992-1996

Year	Age (years)	Obesity I (%)	Obesity II (%)	Parental diabetes (%)
1992	< 40	36.4 vs.24.2	9.1 vs. 12.1	30.0 vs. 60.6
	40-49	37.9 vs. 38.0	6.1 vs. 2.6	53.2 vs. 52.4
	50-59	39.3 vs. 33.7	9.3 vs. 4.6	38.6 vs. 40.9
	60-69	37.1 vs. 36.6	6.4 vs. 4.2	20.5 vs. 22.8
	≥70	37.5 vs. 32.6	4.8 vs. 1.9	8.5 vs. 10.5
				
1993	< 40	36.8 vs. 48.7	5.3 vs. 10.3	52.6 vs. 65.7
	40-49	45.8 vs. 41.7	12.0 vs. 6.0	50.6 vs. 48.4
	50-59	44.7 vs. 37.5	4.8 vs. 3.6	33.7 vs. 39.3
	60-69	39.4 vs. 37.9	5.1 vs. 3.5	23.9 vs. 22.1
	≥70	33.0 vs. 32.4	4.2 vs. 2.7	8.9 vs. 8.6
				
1994	< 40	58.1 vs. 42.6	19.4 vs. 11.8	65.5 vs. 50.0
	40-49	47.6 vs. 43.2	11.8 vs. 8.6	47.8 vs. 50.4
	50-59	46.9 vs. 39.3*	5.4 vs. 6.0	32.2 vs. 36.2
	60-69	39.4 vs. 41.6	6.9 vs. 5.6	19.6 vs. 19.4
	≥70	47.5 vs. 38.1	5.1 vs. 4.4	11.2 vs. 7.9
				
1995	< 40	52.1 vs. 44.4	8.3 vs. 15.9	55.6 vs. 48.8
	40-49	43.5 vs. 44.3	10.1 vs. 8.3	46.8 vs. 49.5
	50-59	53.1 vs. 38.8*	7.5 vs. 5.7	37.2 vs. 34.5
	60-69	39.8 vs. 42.2	5.8 vs. 5.0	16.5 vs. 19.9
	≥70	41.7 vs. 36.8	6.0 vs. 3.0	9.0 vs. 6.2
				
1996	< 40	63.0 vs. 61.8	18.5 vs. 22.5	44.8 vs. 41.4
	40-49	56.0 vs. 48.0	10.9 vs. 9.5	46.7 vs. 42.8
	50-59	49.4 vs. 47.1	11.3 vs. 6.0*	39.1 vs. 35.2
	60-69	45.0 vs. 44.1	10.0 vs. 5.6*	23.2 vs. 17.4
	≥70	30.1 vs. 37.7	4.8 vs. 5.2	1.4 vs. 8.2*

Obesity I and II represent body mass index ≥25 and ≥30 kg/m^2^, respectively.

Data are expressed as ever chewers vs. never-chewers with significant *p *values (< 0.05) indicated by asterisks, otherwise *p *values are > 0.05.

## Discussion

### Higher risk of T2DM in betel chewers

The findings of this study confirmed the link between betel nut chewing and the development of T2DM as observed in a previous population-based prevalence study in Taiwan [8]. The age-specific incidence of newly diagnosed T2DM increased with age reaching a peak in the age group of 60-69 years in ever chewers but kept on increasing at higher ages in the never-chewers (Table 3). One of the reasons for such a decline in T2DM incidence in the eldest age group aged ≥70 years among chewers was that chewers might have died young due to betel chewing related illness. Another reason was that most chewers would have developed T2DM at an earlier age that peaked at 60-69 years (Table 3).

Because the prevalence rates of betel nut chewing in the general population were surveyed during the years 1999-2001 [8] and these prevalence rates would probably be higher than the actual prevalence rates within the years 1992-1996 in the present study, the use of these data would only overestimate the population numbers of ever chewers and underestimate the population numbers of never-chewers. This would result in an underestimation of incidence rates in ever chewers and overestimation of incidence rates in never-chewers, meaning that the incidence rate ratios of newly diagnosed T2DM would be underestimated.

Although the incident case numbers were adjusted by a correction factor of 5.74, one concern is how accurate is this factor for the completeness of identification of all incident cases. However, even if this correction factor was not accurate, the incidence rate ratios would not be affected, because simultaneously multiplying a correction factor in the denominator and numerator would not affect the calculation.

### Dose-responsiveness and strength of association

Because of the lack of data on the duration of betel nut chewing and the amount of betel nuts used per day, it was not possible to evaluate the relationship between exposure dosage and the incidence of newly diagnosed diabetes in this study. However, the increasing incidence rate ratios with age (except for the eldest age group of ≥70 years, Table 3) could also be viewed as a dose-response relation between betel nut chewing and incidence of newly diagnosed T2DM, if the chewing habit in most subjects was developed during adolescence and at younger ages. The older they were, the longer the exposure duration would have been. The strength of association as indicated by the incidence rate ratios (Table 3) was also strong enough to suggest a cause-effect relation between betel chewing and T2DM.

Because the information on the habit of betel nut chewing was collected at the time of questionnaire administration, it leaves open the possibility of the habit being recent in some subjects. This might be a weakness of the study. However, epidemiologic studies in Taiwan suggested that such a habit mostly developed during adolescent and in young adults [6,7,32-34]. Therefore, this potential weakness is unlikely to affect the findings of the study.

### Consistency in findings

The findings were consistent for incidence rates throughout the years 1992-1996 (Table 3) and for analyses of both prevalent cases of T2DM (Table 2) and incident cases of newly diagnosed T2DM (Table 3). The magnitude of the odds ratio obtained from all prevalent cases in the present study of 1.53 (1.45-1.61) was very comparable to the odds ratio of 1.41 (1.18-1.68) obtained by Tung et al. [8]. The age-specific odds ratios estimated from prevalent cases (Table 2) were much smaller than those of the incidence rate ratios obtained from incident cases (Table 3) in the older age groups, suggesting a confounding effect of attenuation caused by attrition from mortality in the older age groups.

### Temporal correctness

It should be noted that in the prevalence analyses (Table 2), we did not know whether subjects started betel nut chewing before or after T2DM onset but in incidence analyses (Table 3) the data were available from the time of questionnaire administration. Because the chewing habit begins early, long before the diagnosis of incident T2DM between 1992 and 1996, the correctness of temporality in these patients is certain.

### Comparable incidence of T2DM in chewers and other high risk groups

Incidence rates of T2DM in chewers aged 60-69 years was at least 10% per year (Table 3). This might suggest that most of the chewers would have developed diabetes after 10 years. However this is unlikely since many other factors contribute to incidence. For example, ever chewers aged ≥70 years in the present study had a lower incidence than those aged 60-69 years (Table 3) and the increases in mortality due to other causes or to betel related illness itself, might reduce the incidence of new T2DM in subjects at this age. Furthermore, the fact that T2DM incidence is comparable in three other studies suggests that the present findings are reliable; (1), age-specific T2DM incidence in never-chewers in the present study were similar to those reported in a prospective epidemiologic study during the years 1990-1996 in the general population of Taiwan as assessed by fasting plasma glucose [35]; (2), T2DM incidence diagnosed at 75 g oral glucose tolerance test between 1989 and 1993 in arsenic-exposed subjects (with an increased risk of T2DM) in Taiwan was close to 100/1,000 person-years in those aged 65-74 years [36]; (3), T2DM incidence in high-risk Pima Indians in the USA between 1991 and 2003 was about 70.8/1,000 person-years for the age group 55-64 years [37].

### Strengths of the study

The strengths of this study included the large population-base, the consistency of the findings over 5 years in both prevalence and incidence, the dose-response relationship found and the correctness of temporality between betel nut chewing and newly diagnosed T2DM.

### Limitations of the study

Limitations of the study include the lack of complete data sets for several socioeconomic factors (e.g., jobs, income and education etc.) and lifestyle (e.g., diet, and physical activity) that are possible confounders. However, obesity and parental diabetes are the two most important risk factors for T2DM in Taiwan [26] and we were able to compare the distribution of these two risk factors between ever-chewers and non-chewers in the newly diagnosed diabetic patients (Table 4). Since the data for these risk factors did not differ between the majority of age categories (Table 4) the increased risk of T2DM in ever-chewers could not be ascribed to either of these two major risk factors of diabetes. Whether the association reported is causal can best be answered by prospective follow-up studies. However, such studies require large sample sizes and would not be easy to conduct because of the low T2DM incidence in young people, the apparent need for prolonged exposure to betel nut chewing for T2DM development and the likelihood of high losses to follow-up. Furthermore, in view of the recognized carcinogenicity of the betel chewing habit [38], such trials are unlikely to be acceptable on ethical grounds. Meanwhile, the present study provides further valuable information, derived from data obtained in a large population-based study, that supports the existence of a relationship between exposure to betel nut chewing and increases in both prevalence of T2DM and in the incidence of newly diagnosed cases.

## Conclusions

This study provides useful information on the relation between betel nut chewing and both prevalent and incident T2DM which is consistent over five consecutive years from 1992 to 1996 and that excludes possible confounding by obesity or parental diabetes; the strength of the association found and the correctness of temporality add further support to earlier reports suggesting that the habit of chewing betel nut can be diabetogenic in humans.

## Competing interests

The author declares that they have no competing interests.

## Authors' contributions

CH: Design of the study, data collection, analysis, interpretation of data, and writing the manuscript.
